# Lactate administration induces skeletal *muscle* synthesis by influencing Akt/mTOR and MuRF1 in non‐trained mice but not in trained mice

**DOI:** 10.14814/phy2.15952

**Published:** 2024-02-21

**Authors:** Sunghwan Kyun, Jisu Kim, Deunsol Hwang, Inkwon Jang, Hun‐Young Park, Kiwon Lim

**Affiliations:** ^1^ Department of Sports Medicine and Science in Graduated School Konkuk University Seoul Korea; ^2^ Physical Activity and Performance Institute (PAPI) Konkuk University Seoul Korea; ^3^ Department of Physical Education Konkuk University Seoul Korea

**Keywords:** exercise, lactate, mTOR pathway, MuRF1, oral administration, protein synthesis

## Abstract

The perception regarding lactate has changed over the past decades, and some of its physiological roles have gradually been revealed. However, the effects of exogenous lactate on skeletal muscle synthesis remain unclear. This study aimed to confirm the effects of a 5‐week lactate administration and post‐exercise lactate administration on skeletal muscle synthesis. Thirty‐two Institute of Cancer Research mice were randomly assigned to non‐trained + placebo, non‐trained + lactate, trained + placebo, and trained + lactate groups. Furthermore, 3 g/kg of lactate or an equivalent volume of saline was immediately administered after exercise training (maximum oxygen uptake: 70%). Lactate administration and/or exercise training was performed 5 days/week for 5 weeks. After the experimental period, it was observed that lactate administration tended to elevate skeletal muscle weight, increased protein kinase B (*p* < 0.05) and mammalian target of rapamycin (*p* < 0.05) mRNA levels, and decreased muscle ring‐finger protein‐1 expression (*p* < 0.05). Lactate administration after exercise training significantly enhanced plantaris muscle weight; however, it had no additional effects on most signaling factors. This study demonstrated that a 5‐week lactate administration could stimulate skeletal muscle synthesis, and lactate administration after exercise training may provide additional effects, such as increasing skeletal muscle.

## 1 INTRODUCTION

Skeletal muscles can be quantitatively altered by the balance between protein synthesis and degradation, which is affected by various factors (Anthony, 2016; Atherton & Smith, 2012). Exercise is a widely accepted strategy for enhancing skeletal muscle by upregulating protein synthesis (Kumar et al., 2009; Tipton & Wolfe, 2001). Previous studies have shown that exercise training induces muscle hypertrophy by increasing protein synthesis signaling, such as the protein kinase B (Akt)/mammalian target of the rapamycin (mTOR) pathway (Bodine, 2006; Devin et al., 2016). Furthermore, this signaling can block E3 ubiquitin ligases (muscle ring‐finger protein‐1 [MuRF1] and muscle‐specific F‐box protein [MAFbx]), thus suppressing atrophy and protein degradation (Latres et al., 2005; Stitt et al., 2004; Zeng et al., 2020). Therefore, exercise training has been confirmed to induce skeletal muscle synthesis; however, the trigger for exercise‐induced muscle synthesis has not been fully elucidated.

During exercise, various metabolic intermediate and bioactive molecules are secreted from skeletal muscles (Hargreaves & Spriet, 2020; Leal et al., 2018). Among them, lactate has been recognized as a “fatigue‐inducing molecule” because early studies have shown that lactate induces acidosis and contributes to a decreased exercise capacity (Hollidge‐Horvat et al., 2000; Sutton et al., 1981). However, recent studies have confirmed that acidosis associated with exercise is induced by increased H+ production from adenosine triphosphate hydrolysis (Robergs et al., 2004). In addition, lactate is used as an energy resource that can transit between body compartments through the Cori cycle between the muscles and liver and from cell to cell or between intracellular sites through the lactate shuttle (Brooks, 2002; Hashimoto & Brooks, 2008). Recent research has changed the perception of lactate, leading to new possibilities for its physiological role.

Exercise training, which produces lactate (Gollnick et al., 1986), is acknowledged as a strategy to further increase skeletal muscle synthesis and related signaling (Atherton & Smith, 2012; Gholipour et al., 2022; Pasiakos et al., 2010). Similarly, previous studies have demonstrated that exercise training with hypoxia or blood flow restriction, in which lactate production is artificially upregulated (Teixeira et al., 2018; Wang et al., 2018), increases muscle synthesis and strength compared with a similar intensity exercise in a normal state (Feriche et al., 2017; Karabulut et al., 2010; Sudo et al., 2017). Furthermore, several cell studies on lactate have confirmed the effects of increased lactate concentrations on muscle synthesis and cell cycle. Liu et al. (2023) demonstrated that lactate accumulated during cell proliferation regulates cell cycle and proliferation via sentrin‐specific protease 1. Li et al. (2014) showed that increased lactate concentration in CHO‐K1 cells decreased the accumulation of cyclic adenosine monophosphate, which inhibits mTOR, and Ohno et al. (2019) reported that the higher the lactate concentration (0–20 mM), the larger the myotube diameter of C2C12 cells in mice. Therefore, lactate may partially affect the mechanism of exercise‐induced muscle synthesis.

Motivated by lactate's relatively novel physiological roles, recent studies have investigated the effects of exogenous lactate on skeletal muscles. Cerda‐Kohler et al. (2018) reported that an injection of 3 g/kg of lactate in mice increased blood lactate concentration (approximately 20 mM) after 5–15 min and upregulated the phosphorylation of Akt (Thr308 and Ser473) and ribosomal protein S6 kinase beta‐1 (P70S6K) in Type 2 muscles after 40 min. In addition, our previous study demonstrated that oral administration of 2 g/kg of sodium lactate increased mRNA levels of *insulin growth factor (IGF) receptor, Akt*, and *mTOR* in the plantaris muscle after 30–60 min but did not affect the degradation factors (MuRF1 and MAFbx) (Kyun et al., 2020). These previous studies collectively highlight lactate's benefit in enhancing skeletal muscle synthesis. Furthermore, these suggest that lactate administration after exercise training can have additional effects on skeletal muscle synthesis by increasing blood lactate more (Jang et al., 2021). However, Liegnell et al. (2020) reported that exogenous lactate infusion during resistance exercise did not increase protein synthesis in human skeletal muscle. Furthermore, Nordström et al. (2023) showed that lactate infusion at rest increased the lactate receptor of G protein‐coupled receptor 81 (GPR81) activation, but lactate infusion during resistance exercise had no effects on GPR81. These previous studies suggest that lactate treatment during exercise has potential to inhibit the anabolic effect of lactate, and acute lactate treatment may have less effect on protein synthesis than long‐term treatment.

Nevertheless, only few studies have investigated the effects of a 5‐week oral lactate administration, and previous studies have not directly investigated the effect of lactate administration after exercise training on skeletal muscle synthesis. Therefore, the present study had two purposes: (a) to determine the effects of a 5‐week oral lactate administration on skeletal muscle and protein synthesis (Akt/mTOR pathway) and degradation factors (E3 ubiquitin ligases) and (b) to confirm whether lactate administration after exercise training has additional effects on skeletal muscle synthesis.

## 2 MATERIALS AND METHODS

### 2.1 Animal care

Ethical approval for this study was obtained from the Konkuk University Institutional Animal Care and Use Committee (No. KU19149). The animal experiment was strictly conducted as per the ethical guidelines of the Animal Experiment Research Center of Konkuk University. The methods of animal experiment were performed in accordance with ARRIVE guidelines. Six‐week‐old male Institute of Cancer Research mice (*n* = 32) were obtained from Orient Bio (Seongnam, Korea) and assigned to standard cages (four mice/cage). Circadian (12:12‐h light–dark cycle), humidity (50%), and temperature (23 ± 1°C) conditions were controlled by automatic controller to maintain normal conditions. A standard commercial diet (5L79, Orient Bio, Seongnam, Korea) and water were provided ad libitum. Food intake and body weight were measured daily between 9 and 10 a.m. to determine changes by lactate administration.

### 2.2 Study design

A pilot experiment was conducted to confirm whether oral lactate administration can increase blood lactate. The blood lactate concentration in mice (*n* = 6) after oral lactate administration was measured from the blood obtained from their tails using Lactate Pro2 (Arkray Inc., Kyoto, Japan). Lactate (sodium lactate, 195‐05965, Wako Chemical, Osaka, Japan) was orally administered using oral gavage at 3 g/kg (Cerda‐Kohler et al., 2018; Jang et al., 2021). The blood lactate was measured at nine time points (before administration and 15, 30, 45, 60, 90, 120, 150, and 180 min after administration).

**FIGURE 1:**
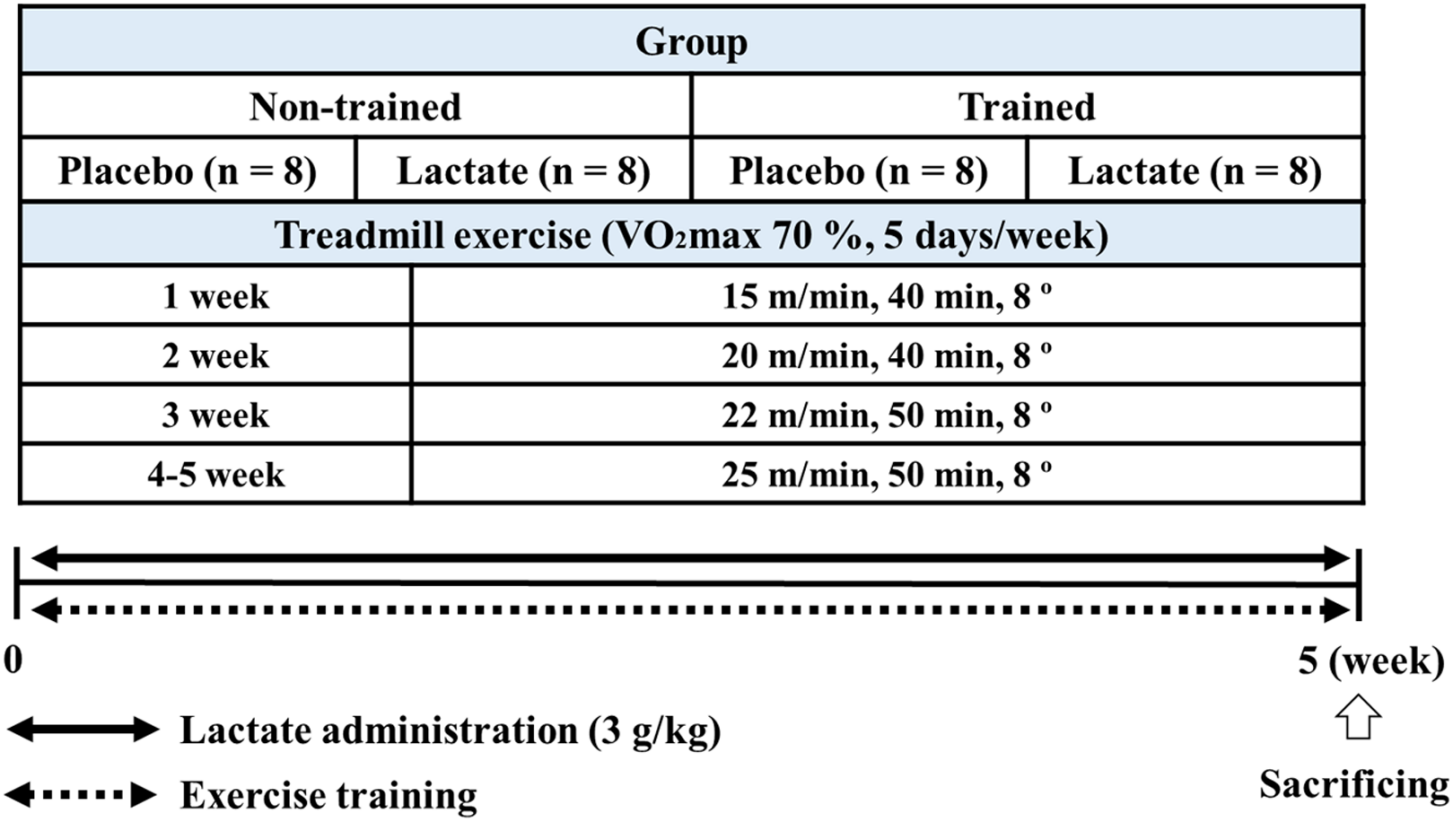
Study design. Study design of 5 weeks lactate administration and post‐exercise lactate administration.

In the present study, the mice were randomly divided into four groups: non‐trained + placebo (Non/Pla; *n* = 8), non‐trained + lactate (Non/Lac; *n* = 8), trained + placebo (Tr/Pla; *n* = 8), and trained + lactate (Tr/Lac; *n* = 8). Exercise training intensity was set at the “lactate threshold” (under 70% maximal oxygen uptake [VO_2max_]) based on a previous study and was gradually increased (first week; 15 m/min, 40 min, 8°, second week; 20 m/min, 40 min, 8°, third week; 22 m/min, 50 min, 8°, fourth and fifth week 25 m/min, 50 min, 8°) (Hwang et al., 2020). Lactate and placebo groups received 3 g/kg of sodium lactate and same volume of saline solution, respectively. Lactate or saline was administered using oral gavage immediately after each exercise training. Exercise training and lactate administration were performed 5 days/week from 10 to 11 a.m. Mice were sacrificed using intraperitoneal anesthesia with 10 μL/g of 1.25% avertin 48 h after the last exercise training and lactate administration. Blood (serum) and skeletal muscles (plantaris and extensor digitorum longus [EDL]) were collected immediately after sacrifice. The study design is shown in Figure 1.

### 2.3 Blood analysis

Blood samples were immediately obtained after intraperitoneal anesthesia and centrifuged at 1500*g* for 15 min under 4°C before collecting the supernatant (serum). After blood sample preparation, serum concentrations of lactate (KTB1100, Abbkine, Wuhan, China) and glucose (K039‐H1, ARBOR ASSAYS, Ann Arbor, MI, USA) were measured using a colorimetric kit according to the manufacturer's protocol and were assessed using colorimetric detection at 450 and 560 nm, respectively.

### 2.4 mRNA analysis

Reverse‐transcription polymerase chain reaction (RT‐PCR) was used to measure the mRNA levels of protein synthesis and degradation factors in the plantaris muscle. Total RNA was extracted using 1 mL of TRIzol reagent (79306; Qiagen, Hilden, Germany), and 200 μL chloroform (038‐02601, Wako Chemical, Osaka, Japan) was then added. After centrifuging the sample at 12,000*g* for 15 min under 4°C, the supernatant was separated into another tube, and 200 μL isopropanol (d2377, DUKSAN, Seoul, Korea) was added before mixing and reacting. The samples were centrifuged at 12,000*g* for 15 min under 4°C to collect RNA pellets. RNA pellets were diluted in 30 μL of diethyl pyrocarbonate water and heated at 55°C. AmfiRivert complementary DNA (cDNA) Synthesis Platinum Master Mix (R5600; GenDEPOT, Katy, TX, USA) was used to synthesize cDNA from the extracted total RNA according to the manufacturer's instructions. cDNA was amplified using amfiEco Taq DNA polymerase (P0701; GenDEPOT, Katy, TX, USA) according to the manufacturer's instructions: 1 μL of cDNA with 24 μL of Taq DNA polymerase, 2× reaction buffer, PCR water, and forward and reverse primers. Detailed primer sequences and qRT‐PCR cycling conditions for each gene are presented in Table S1. The PCR products were separated using 1% agarose gels composed of 1× TAE buffer (50 mL), 0.5 g LE agarose, and 1.25 μL Safe‐Pinky DNA gel staining solution (S1001‐025; GenDEPOT, Katy, TX, USA). PCR product bands were analyzed using Print Graph 2M (ATTO, Sungnam, Korea) and normalized to GAPDH.

### 2.5 Protein analysis

The EDL muscle was analyzed using western blotting to confirm the protein expression of muscle synthesis and degradation factors. The EDL muscles were homogenized in 250 μL of protein extraction buffer (EzRIPA Lysis kit, ATTO, Tokyo, Japan). Whole lysates were centrifuged at 14,000*g* for 15 min under 4°C, and the protein concentration of supernatants transferred into new tubes was determined using a BCA assay kit (Thermo Fisher Scientific, Waltham, MA, USA). After samples were denatured by heating at 100°C for 5 min, total protein was separated using 10% SDS‐PAGE and transferred to polyvinylidene difluoride membranes (Millipore, Billerica, MA, USA). The membranes were blocked for 1 h with 5% skim milk (Difco, Franklin Lakes, NJ, USA) and washed four times for 5 min using phosphate‐buffered saline (PBS) with 0.1% Tween 20 (PBS‐T). The membranes were incubated overnight (16–24 h) at 4°C with 3% skim milk and primary antibodies against GAPDH (1:1000, sc‐35062, Santa Cruze Biotechnology, USA), IGF receptor (1:1000, #3027, Cell signaling technology, USA), Akt (1:1000, #4691, Cell signaling technology, USA), p‐Akt (1:1000, #4058, Cell signaling technology, USA), mTOR (1:1000, #2983, Cell signaling technology, USA), p‐mTOR (1:1000, #5536, Cell signaling technology, USA), P70S6K (1:1000, #2708, Cell signaling technology, USA), p‐P70S6K (1:1000, #9205, Cell signaling technology, USA), MuRF1 (1:1000, sc‐3998608, Santa Cruze Biotechnology, USA), MAFbx (1:1000, sc‐166,806, Santa Cruze Biotechnology, USA), and Forkhead box protein O1 (FoxO1) (1:1000, #9461, Cell signaling technology, USA). After washing with PBS‐T, the membranes were incubated with a horseradish peroxidase‐conjugated secondary antibody (anti‐mouse, sc‐516102; Santa Cruz Biotechnology, USA; anti‐rabbit, sc‐2357, Santa Cruz Biotechnology, USA) with 3% skim milk for 1 h at room temperature (24–25°C). Immunodetection was performed using ECL reagent (Amersham Biosciences, Uppsala, Sweden). Images showing the results of the quantitative analysis were assessed using Image J software (NIH Image Engineering, Bethesda, MD, USA).

### 2.6 Statistical analysis

All data were analyzed using IBM SPSS Statistics version 25 (IBM Corp., Armonk, NY, USA) software. The significant main effects (supplement [placebo vs. lactate] and exercise [non‐trained vs. trained]) and interactions were analyzed using a two‐way analysis of variance, followed by a post hoc test for the least significant difference. Statistical significance was set at *p* < 0.05. Data were expressed as mean ± standard deviations.

## 3 RESULTS

### 3.1 Effects of acute lactate administration on blood lactate

Blood lactate after acute lactate administration is presented in Table 1. Blood lactate level significantly increased from 3.03 ± 0.31 mmol/L to 8.22 ± 2.38 mmol/L after 15 min of lactate administration and returned to the baseline level (approximately 3.67 ± 1.17 mmol/L) after 3 h. Therefore, exogenous lactate administration increases blood lactate concentration.

**TABLE 1:** Blood lactate concentration after acute oral lactate administration.

	Time after lactate administration (min)								
	0	15	30	45	60	90	120	150	180
Lactate (mmol/L)	3.0 ± 0.3	8.2 ± 2.4*	6.7 ± 0.8*	6.5 ± 1.2*	5.7 ± 0.3*	5.7 ± 1.2*	4.9 ± 0.7*	5.2 ± 0.8*	3.7 ± 1.1

*Note*: Parameters of lactate concentration during the resting period after oral lactate administration. 0 min indicates before lactate administration, whereas 15, 30, 45, 60, 90, 120, 150, and 180 min indicate after lactate administration. Data are presented as mean ± standard deviation.

* *p* < 0.05 vs. 0 min.

### 3.2 Effects of 5‐week lactate administration and post‐exercise lactate administration on body weight, food intake, and skeletal muscle weight

Body weight, food intake, and skeletal muscle weight after a 5‐week lactate administration and post‐exercise lactate administration are presented in Table 2. After the experiment, body weight did not differ among the groups. However, food intake demonstrated main effects (supplement, *p* = 0.000; and exercise, *p* = 0.014) and interactions (*p* = 0.002). Furthermore, body weight was significantly lower in the Non/Lac group than in the Non/Pla group (*p* < 0.05), whereas it was significantly higher in the trained groups than in the non‐trained groups (Non/Pla vs. Tr/Pla, *p* < 0.05; Non/Lac vs. Tr/Lac, *p* < 0.05). The muscle weight of two different skeletal muscles was measured. The only supplement effect was observed in plantaris and EDL muscles (*p* = 0.03 and *p* = 0.029, respectively). The plantaris muscle weight was significantly higher in the Tr/Lac group than in the Tr/Pla group (*p* < 0.05). However, the EDL muscle weight was not significantly different among the groups.

**TABLE 2:** Body weight, food intake, and skeletal muscle weight.

	Non‐trained		Trained		*p*‐value
	Placebo	Lactate	Placebo	Lactate	
Body weights (g/mouse)	35.93 ± 0.78	34.64 ± 0.93	35.15 ± 0.64	35.47 ± 0.72	Supplement 0.966 Exercise 0.387 Interactions 0.152
Food intake (g/5 week)	121.22 ± 2.1	109.08 ± 2.0*	147.28 ± 3.4^#^	148.87 ± 2.3^#^	Supplement 0.000 Exercise 0.014 Interactions 0.002
Plantaris (mg/g)	18.6 ± 2.2	19.8 ± 1.75	18.6 ± 1.19	20.1 ± 1.13*	Supplement 0.03 Exercise 0.747 Interactions 0.747
Extensor digitorum longus (mg/g)	11.5 ± 0.93	12.5 ± 1.51	12.4 ± 1.6	13.6 ± 1.41	Supplement 0.029 Exercise 0. 051 Interactions 0.8

*Note*: Data are presented as mean ± standard deviation.

* *p* < 0.05, placebo vs. lactate.

^#^ *p* < 0.05, non‐trained vs. trained.

### 3.3 Effects of 5‐week lactate administration and post‐exercise lactate administration on blood lactate and glucose

The serum levels of lactate and glucose were measured to determine whether they were affected by the 5‐week lactate administration and post‐exercise lactate administration (Table 3). After 3 h of fasting, blood samples were obtained from inferior vena cava. The serum lactate concentration did not significantly differ among the groups. The serum glucose concentration demonstrated only an exercise effect (*p* = 0.004), and there was a significant difference between the Tr/Pla and Tr/Lac groups (*p* < 0.05).

**TABLE 3:** Blood concentration of lactate and glucose.

	Non‐trained		Trained		*p*‐value
	Placebo	Lactate	Placebo	Lactate	
Lactate (mmol/L)	3.71 ± 0.60	3.29 ± 0.72	3.18 ± 0.29	3.15 ± 0.55	Supplement 0.265 Exercise 0.101 Interaction 0.332
Glucose (mg/dL)	166.8 ± 20.6	155.4 ± 23.5	131.1 ± 32.0*	135.5 ± 21.3	Supplement 0.691 Exercise 0.004 Interaction 0.375

*Note*: Data are presented as mean ± standard deviation.

* *p* < 0.05, non‐trained vs. trained.

### 3.4 Effects of 5‐week lactate administration and post‐exercise lactate administration on mRNA levels of protein synthesis and degradation factors in plantaris muscles of mice

**FIGURE 2:**
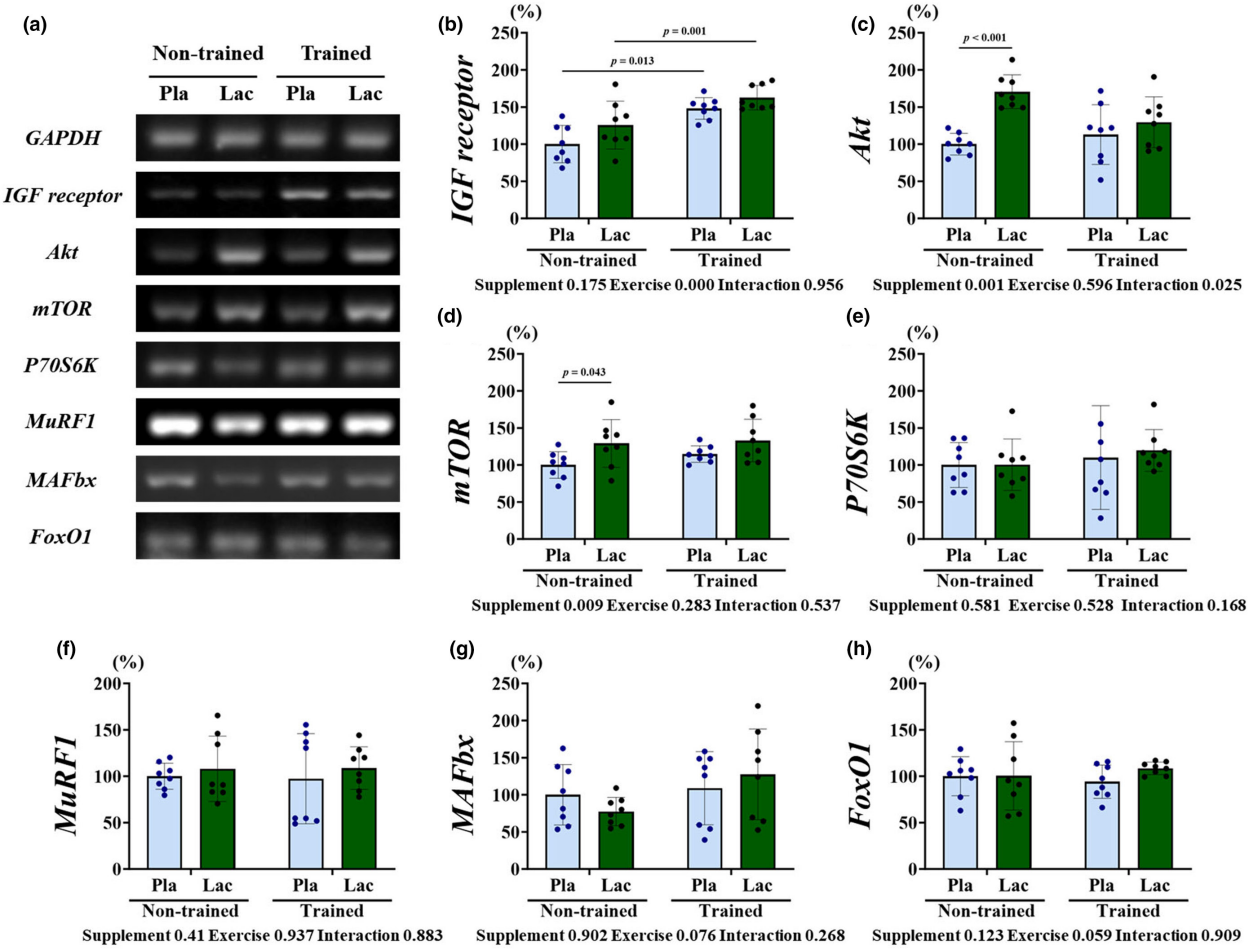
mRNA expression of protein synthesis and degradation factors in the plantaris muscle. (a) Polymerase chain reaction band. (b) *IGF receptor*, (c) *Akt*, (d) *mTOR*, (e) *P70S6K*, (f) *MuRF1*, (g) *MAFbx*, and (h) *FoxO1* expression levels were normalized by GAPDH. Results are presented as means ± standard deviation. Pla, placebo (saline administration); Lac, lactate administration.

RT‐PCR analysis was performed to investigate the mRNA expression of protein synthesis and degradation factors. The mRNA levels of *IGF receptor*, *Akt*, *mTOR*, and *P70S6K* were measured to confirm alternation protein synthesis signaling (Figure 2b–e). Only the *IGF receptor* demonstrated the main effect of exercise (*p* = 0.000). Furthermore, the mRNA of *Akt* exhibited the main effect of supplementation (*p* = 0.001) and interactions (*p* = 0.025), which were significantly upregulated in the Non/Lac group than in the Non/Pla group (*p* < 0.05). Furthermore, *mTOR* demonstrated only the main effect of supplementation (*p* = 0.009), which was significantly upregulated in the Non/Lac group than in the Non/Pla group (*p* < 0.05). The mRNA level of *mTOR* was increased in the Non/Pla group; however, the *P70S6K* of mTOR downregulation was not significantly different among the groups. The present study measured the mRNA levels of *MuRF1*, *MAFbx*, and *FoxO1* to confirm changes in protein degradation factors after the 5‐week lactate administration and post‐exercise lactate administration (Figure 2f–h), revealing that the mRNA levels of protein degradation factors were not affected.

### 3.5 Effects of 5‐week lactate administration and post‐exercise lactate administration on protein expression of protein synthesis and degradation factors in EDL muscle of mice

**FIGURE 3:**
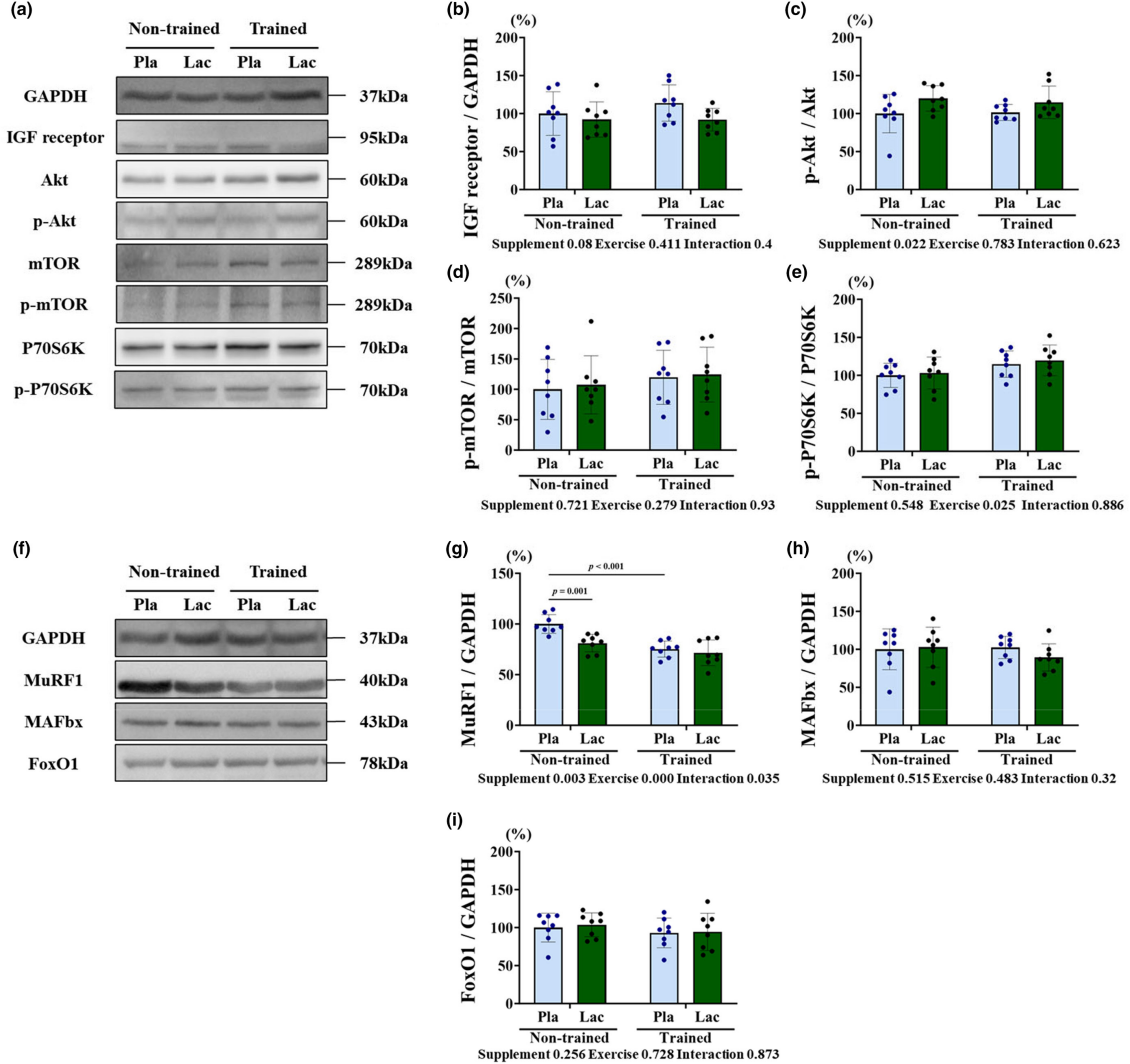
Protein expression of protein synthesis and degradation factors in EDL muscle. (a) and (f) present the western band of protein synthesis and degradation factors, respectively. (b) IGF receptor, (c) Akt, (d) mTOR, (e) P70S6K, (g) MuRF1, (h) MAFbx, and (i) FoxO1 represent protein expression. Results are presented as means ± standard deviation. Pla, placebo (saline administration); Lac, lactate (lactate administration).

Western blotting was performed to elucidate the effects of the 5‐week lactate administration and post‐exercise lactate administration on protein expression of protein synthesis (Figure 3b–e) and degradation (Figure 3g–i) factors. The protein expression of the synthesis factors of the IGF receptor, Akt phosphorylation, and mTOR phosphorylation was not significantly affected. However, P70S6K phosphorylation was the only main effect of exercise (*p* = 0.025). Regarding the protein expression of skeletal muscle degradation factors, MAFbx and FoxO1 were not affected by the 5‐week lactate administration and post‐exercise lactate administration. However, the MuRF1 expression demonstrated the main effects (supplementation, *p* = 0.003; and exercise, *p* = 0.000) and interactions (*p* = 0.035), which were significantly downregulated in the Non/Lac and Tr/Pla groups than in the Non/Pla groups (*p* < 0.05, respectively).

## 4 DISCUSSION

The effects of exogenous lactate administration have been investigated, whereas those of oral lactate administration on muscle synthesis have not been elucidated. The present study investigated the effects of a 5‐week lactate administration on skeletal muscle synthesis and the additional effects of lactate administration after exercise training. The main findings were that the 5‐week lactate administration positively influenced the expression of protein synthesis (mRNA levels of *Akt* and *mTOR*) and degradation (MuRF1 protein expression) factors. Furthermore, lactate administration after exercise training increased skeletal muscle weight but did not affect Akt/mTOR signaling and E3 ubiquitin ligases.

The present study showed that lactate administration decreased food intake by 11.1% and decreased body weight. Appetite is regulated by hormones such as leptin and ghrelin (Austin & Marks, 2008). Previous studies have reported that lactate inhibits ghrelin secretion by binding to ghrelin‐producing cells and that central administration of lactate decreases energy intake in rodents (Engelstoft et al., 2013; Lam et al., 2008). Therefore, these studies suggest that oral lactate administration can also reduce food intake by inhibiting ghrelin secretion. However, as the present study did not analyze appetite hormone, analyzing appetite hormones in future studies will be helpful in understanding the effect of oral lactate administration.

Previous studies have reported that lactate activates cell proliferation and increases the myotube diameter and length, myonuclei number, and protein content in cells (Ohno et al., 2018, 2019; Oishi et al., 2015). Furthermore, a study reported that that administration of 1 g/kg of lactate increased blood lactate concentration by 4.1 ± 0.3 mmol/L and that lactate administration for 5 days/week increased the weight of the tibialis anterior muscle after 2 weeks in mice (Ohno et al., 2019). Therefore, we hypothesized that a 5‐week lactate administration would increase skeletal muscle weight. As expected, this study's results showed that acute lactate administration increased the blood lactate concentration and that lactate administration positively affected plantaris and EDL muscles' weight. These findings suggest that increasing blood lactate levels may positively affect skeletal muscle synthesis as part of exercise‐induced muscle synthesis.

The Akt/mTOR pathway and muscle‐specific E3 ubiquitin ligases regulate muscle synthesis and degradation and play important roles in exercise‐induced muscle synthesis (Schiaffino et al., 2013; Schiaffino & Mammucari, 2011). Previous studies primarily focused on the effects of acute lactate intake on protein synthesis and showed that exogenous lactate intake could alter the protein balance by stimulating the Akt/mTOR pathway in Type 2 skeletal muscles (Cerda‐Kohler et al., 2018; Kyun et al., 2020). The present study investigated the effects of oral lactate administration on protein synthesis signaling and confirmed that the mRNA levels of *Akt* and *mTOR* in the plantaris muscle were increased.

While there remains considerable debate regarding the relationship between mRNA levels and protein expression, it is crucial to note their close association. Numerous studies have highlighted a positive correlation between mRNA levels and protein expression (Guo et al., 2008; Wilhelm et al., 2014). In this study, the administration of lactate over a 5‐week period resulted in the upregulation of mRNA levels of Akt and mTOR—factors linked to protein synthesis in skeletal muscle—at a steady state. These findings suggest a potential increase in the protein expression of Akt and mTOR. However, the 5‐week lactate administration did not impact the protein expression in the Akt/mTOR pathway in the EDL muscle. One possible explanation for these outcomes could be that the 5‐week lactate administration period might not have been sufficient for the translation from mRNA to protein expression. Few studies have explored lactate administration for durations exceeding 8 weeks. Thus, it becomes imperative to validate the long‐term effects of lactate administration and to corroborate both mRNA levels and protein expression. Therefore, it is recommended that future studies focus on confirming the correlation between the effects of long‐term lactate administration on mRNA levels and protein expression to ensure more accurate results.

Nevertheless, the present study's results showed that the skeletal muscle weight was positively affected after the 5‐week lactate administration, which was expected as decreasing the concentration of muscle‐specific E3 ubiquitin ligases. MuRF1, an E3 ubiquitin ligase expressed in skeletal muscle, regulates proteolysis (Guo et al., 2008). Moreover, exercise training attenuates protein degradation by downregulating MuRF1 expression in skeletal muscle (Bodine & Baehr, 2014; Zanchi et al., 2009). Interestingly, the present study showed that MuRF1 expression was downregulated by the 5‐week lactate administration. These findings are inconsistent with those of previous our study that acute lactate administration did not affect protein degradation factors (MuRF1 and MAFbx) (Kyun et al., 2020) and suggest that a 5‐week lactate administration may have a different effect on protein degradation factors compared with acute administration. Therefore, the present study confirms that a 5‐week lactate administration can increase skeletal muscle weight by affecting the mRNA levels of protein synthesis factors and protein expression of degradation factor.

Oishi et al. (2015) confirmed that the administration of lactate and caffeine compounds combined with exercise training for 4 weeks increased the weight of the gastrocnemius and anterior tibialis muscles, enhanced the total DNA content, and upregulated anabolic signals in rats. Hashimoto et al. (2019) showed that lactate‐based compounds combined with voluntary running exercises increased the skeletal muscle weight of the plantaris and gastrocnemius muscles in obese rats. The present study also confirmed that the 5‐week lactate administration after exercise training significantly enhanced the plantaris muscle. The present study showed that lactate affects the mRNA levels of *Akt/mTOR* and protein expression of MuRF1; however, lactate administration after exercise training did not result in additional effects.

A possible reason for the increased skeletal muscle caused by lactate administration after exercise training is other factors inducing skeletal muscle synthesis. Previous studies confirmed that lactate activates mitogen‐activated protein kinase kinase (MEK)/ extracellular signal‐regulated protein kinase (ERK) pathway, which stimulates protein synthesis in skeletal muscle (Hashimoto et al., 2019; Schoenfeld, 2010). Li et al. (2014) reported that lactate stimulates hydroxycarboxylic acid receptor‐1, which evokes phosphorylation of ERK, in CHO‐K1 and L6 cells, and Ohno et al. (2019) also reported that lactate increased MEK and ERK phosphorylation in C2C12 cell. Furthermore, Cerda‐Kohler et al. (2018) showed that exogenous lactate injection increased ERK in mice. Collectively previous studies, we supposed that MEK/ERK pathway was activated by lactate administration after exercise training and increased skeletal muscle synthesis. Therefore, a more detailed study investigating the activation of the MEK/ERK pathway is needed to confirm the effects of lactate administration after exercise training on molecular signaling in skeletal muscle synthesis. Nevertheless, because the 5‐week lactate administration after exercise training increased skeletal muscle weight, the present study confirmed that positive results of skeletal muscle synthesis might be observed.

However, a limitation of this study is that although we performed mRNA and protein analysis concerning the increase in skeletal muscle due to lactate administration after exercise training, these analyses do not confirm the effects on myofiber size, cell numbers, or cell differentiation. Therefore, subsequent analytical methods such as cross‐sectional area measurement and immunohistochemistry are considered necessary to more accurately confirm the effects of lactate and exercise on skeletal muscle gain.

## 5 CONCLUSION

The present study confirmed that (a) lactate administration positively affect protein synthesis (mRNA levels of *Akt* and *mTOR*) and degradation (MuRF1 protein expression) and (b) lactate administration after exercise training increased skeletal muscle synthesis as an additional effect. This study did not measure mRNA and protein expression in the same skeletal muscle (mRNA levels in plantaris muscle and protein expression in EDL muscle); however, lactate administration demonstrated the potential to regulate skeletal muscle synthesis positively. Therefore, this study provides new insights into the effects of oral administration of exogenous lactate on skeletal muscle synthesis.

## AUTHOR CONTRIBUTIONS

Conceptualization: S.K., J.K., H.‐Y.P., and K.L.; methodology: S.K., J.K., H.‐Y.P., and K.L.; software, validation, and formal analysis: S.K., J.K., D.H, and H.‐Y.P.; investigation: S.K. and I.J.; writing—original draft preparation: S.K.; writing—review and editing: all authors; visualization: S.K., D.H., and I.J. All authors have read and agreed to the published version of the manuscript.

## FUNDING INFORMATION

This work was supported by the Ministry of Education of the Republic of Korea and the National Research Foundation of Korea (NRF‐2019S1A5B8099542 and NRF‐2021R1G1A1011987). This paper also was written as part of the research support program of Konkuk University for its faculty on sabbatical leave in 2022.

## CONFLICT OF INTEREST STATEMENT

The authors declared no competing interests.

## ETHICS STATEMENT

This study was approved by the Konkuk University Institutional Animal Care and Use Committee (No. KU19149) and strictly conducted as per the ethical guidelines of the Animal Experiment Research Center of Konkuk University.

## Supporting information


Table S1.
