# Supplementary material for: Lactate administration induces skeletal muscle synthesis by influencing Akt/mTOR and MuRF1 in non‐trained mice but not in trained mice
Source: Physiol Rep. 2024 Feb 21;12(4):e15952. doi: 10.14814/phy2.15952 (PMC10881281; doi:10.14814/phy2.15952)
Supplement: Supplementary file 1 — Table S1. [file PHY2-12-e15952-s001.docx]

**Supplementary Table 1. Sequences of mRNA analysis primers.**

| **Gene** | **Sequence** | **Annealing Temperature** | **Cycle** |
| --- | --- | --- | --- |
| *GAPDH* | F–5’ TGC TGG TGC TGA GTA TGT CG 3’ | 54°C | 18 |
|  | R–5’ TGA TGG CAT GGA CTG TGG TC 3’ |  |  |
| *IGF R* | F–5’ TGT GTG TGT CCT GGA TTT GGG 3’ | 58°C | 25 |
|  | R–5’ AGA TTT CTC CAC TCG TCG GC 3’ |  |  |
| *Akt* | F–5’ ATG CTG GAC AAG GAC GGG 3’ | 55°C | 26 |
|  | R–5’ CAC GAT GTT GGC AAA GAA 3’ |  |  |
| *mTOR* | F–5’ ACT GTG TCT TGG CAT CCC TG 3’ | 59°C | 27 |
|  | R–5’ AGC CTT CAG GAT AGG CTC CA 3’ |  |  |
| *P70S6K* | F–5’ GGA AGC GCT CAG CTG TTA GA 3’ | 59°C | 26 |
|  | R–5’ GCC CCC TTT ACC AAG TAC CC 3’ |  |  |
| *MuRF1* | F–5’ GCT GAG CTC CAG TAT CGA GG 3’ | 59°C | 26 |
|  | R–5’ ACG TAC CGA GTC TGG CAG TA 3’ |  |  |
| *MAFbx* | F–5’ GGG GGA AGC TTT CAA CAG 3’ | 55°C | 24 |
|  | R–5’ TGA GGC CTT TGA AGG CAG 3’ |  |  |
| *FoxO1* | F–5’ ACA TTT CGT CCT CGA ACC AGC TCA 3’ | 61°C | 27 |
|  | R–5’ ATT TCA GAC AGA CTG GGC AGC GTA 3’ |  |  |

*GAPDH*, glyceraldehyde 3-phosphate dehydrogenase; *IGF* receptor, insulin-like growth factor receptor; *Akt*, protein kinase B; *mTOR*, mammalian target of rapamycin; *P70S6K*, ribosomal protein S6 kinase beta-1; *MuRF1*, muscle ring-finger protein-1; *MAFbx*, muscle-specific F-box protein; *FoxO1*, forkhead box protein O1.
